# A new answer to old questions

**DOI:** 10.7554/eLife.00515

**Published:** 2013-02-05

**Authors:** Yves Barral

**Affiliations:** 1 **Yves Barral** is at the Institute of Biochemistry, ETH Zurich, Zurich, Switzerlandyves.barral@bc.biol.ethz.ch

**Keywords:** Aging, Metabolism, Oxidative Stress, Redox Regulation, Redox Proteomics, S. cerevisiae

## Abstract

Sudden changes in the level of a coenzyme called NADPH might be the underlying cause of aging in cells.

**Related research article** Brandes N, Tienson H, Lindemann A, Vitvitsky V, Reichmann D, Banerjee R, Jakob U. 2013. Time line of redox events in aging postmitotic cells. *eLife* **2**:e00306. doi: 10.7554/elife.00306

**Image** Exploring aging in yeast

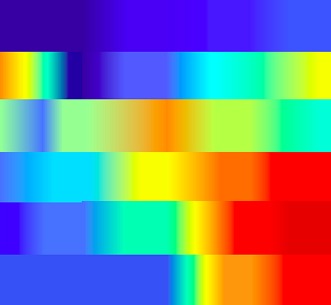

One prominent theory of aging proposes that the physiological decline associated with old age is the result of protein oxidation and oxidative damage, mainly caused by reactive oxygen species. There are various lines of evidence that support this idea: for example, yeast cells that overexpress superoxide dismutase—an enzyme that transforms superoxides, which are a particularly aggressive class of reactive oxygen species, into oxygen and hydrogen peroxide—have extended lifespans (Harris et al., 2003), whereas cells that contain little or no superoxide dismutase live for relatively short times (Longo et al., 1996). Furthermore, aging cells have been shown to accumulate carbonylated proteins (proteins in which certain amino acids have been oxidized by reactive oxygen species), which suggests that oxidative damage does indeed take place during aging and may, therefore, also be one of the causes of aging (reviewed in Nyström, 2005).

However, there are many questions about the links between aging and oxidation (which is the removal of one or more electrons from a chemical species) that remain unanswered: how much protein oxidation takes place during the aging of single cells? What are the main targets of oxidation? And how does oxidation correlate temporally with aging? Answering these questions will clarify whether oxidation actually contributes to aging, or if it merely correlates with the physiological decline observed during aging. Writing in *eLife*, Ursula Jakob and co-workers at the University of Michigan—including Nicolas Brandes and Heather Tienson as joint first authors—supply answers to some of these questions (Brandes et al., 2013).

The Michigan group started by measuring the oxidation status of an amino acid (cysteine) in almost 300 different proteins in yeast cells that had stopped dividing and started the process of chronological aging. The measurements were made over a period of 7–10 days in most cases, and for up to 20 days in some cases. The use of such a large number of proteins allowed the researchers to probe all the different organelles found in yeast cells and a large variety of cellular functions. In parallel, they measured the levels of thioredoxin and glutathione—antioxidants that prevent the oxidation of other molecules and proteins. (Both of these antioxidants also contain cysteine). Finally, they monitored the levels of adenosine triphosphate, which transfers energy within cells, and both nicotinamide adenine dinucleotide phosphate (NADP+) and its reduced version, which is known as NADPH. NADPH is a source of electrons (which means that it is a reducing agent) that is involved in various anabolic reactions and in redox control (that is, maintaining the balance between oxidation and reduction, which is the addition of one or more electrons to a chemical species). These measurements were performed under a number of different dietary regimes, including one—caloric restriction (that is, a reduced calorie intake)—that is known to prolong the lifespan of post-mitotic yeast cells and many other organisms (see Figure 1).

**Figure 1.**
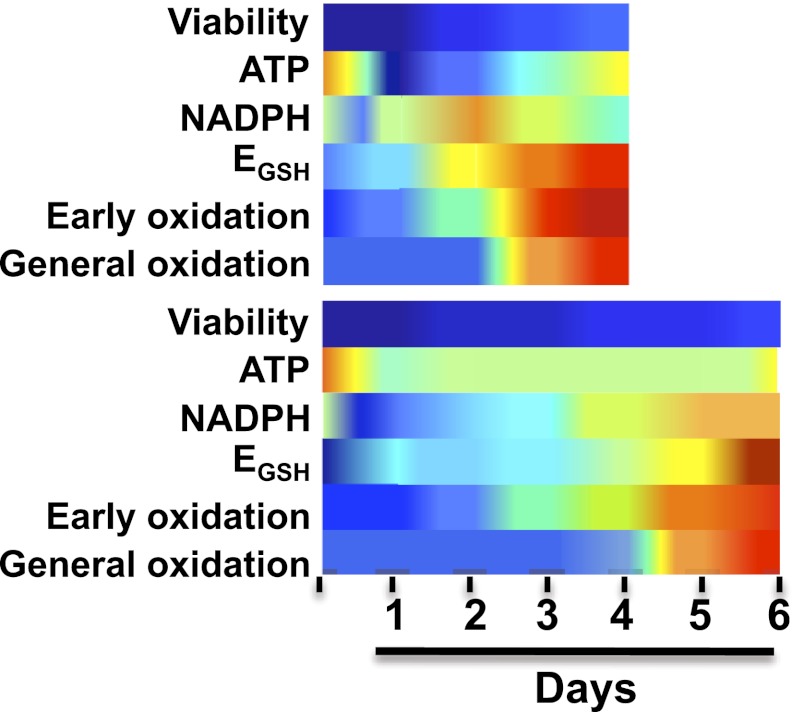
Brandes et al. made a series of measurements every 24 hours on yeast cells under various dietary regimes, including a standard diet (top) and caloric restriction (bottom). As expected, the yeast on the caloric restriction diet lived longer. However, in both regimes the levels of the coenzyme NADPH rose rapidly (dark blue represents high levels of NADPH) and then reduced rapidly (yellow and red represent low levels of NADPH); this was followed by an increase in the oxidation of a small number of ‘early oxidation’ proteins (blue and green represent low oxidation levels; yellow and red represent high levels), which was followed by the oxidation of most of the other proteins measured. Brandes et al. propose that a sudden and substantial decrease in NADPH levels is likely to be the underlying cause of aging, rather than the accumulation of reactive oxygen species. E_GSH_ is the glutathione potential; see figure 7 of Brandes et al. for further information.

As expected, Brandes, Tienson and co-workers observed that oxidation did increase with aging, reaching its maximum value about 24 hours before the viability of the cells started to decrease. This is consistent with the possibility that oxidation has a deleterious effect on cell viability, although it also suggests that this effect is indirect (otherwise the drop in viability would happen immediately, rather than 24 hours after oxidation reaches a maximum). The Michigan group then showed that the proteins responded to oxidation in one of three ways. About 10% of the proteins—including the enzyme that reduces thioredoxin—are oxidized 48 hours before the decline of viability. Another 80% or so of the proteins are oxidized about 24 hours before mortality increases, with the remaining proteins being only minimally affected by aging.

The vast majority of the proteins that are particularly sensitive to oxidation by reactive oxygen species are in the group that are oxidized 24 hours before mortality increases, which means that these species are not the primary cause of the oxidation that is associated with aging in yeast. Rather, the data are consistent with a scenario in which a collapse in NADPH levels causes the oxidation of proteins involved in redox control. This first wave of oxidation causes a second wave in which the oxidation propagates to most (but not all) of the proteins. A small number of proteins are protected from oxidation, and a better understanding of the mechanisms responsible for this protection might help explain how cells can remain viable for 24 hours after the majority of proteins have been oxidized. The same sequence of events happens during caloric restriction, but over a longer timescale.

As Brandes, Tienson et al. remark, it is striking that the oxidation events they detect are reversible. This observation is consistent with the suggestion that an important role of many of the cysteines in yeast cells might be to buffer changes in the redox potential of the cell (Gallogly and Mieyal, 2007; Hansen et al., 2009). The redox potential, which is measured in volts, is essentially a measure of electron concentration. In this view, therefore, oxidation events (and the damage they cause) are essentially a by-product of the proteome's role in sensing and adjusting the global redox potential of the cell. Indeed, the Michigan team goes further and suggests that some of these cysteines might have been selected to help regulate (depending on the redox potential) the function of the proteins in which they are embedded. Thus, the work of Brandes, Tienson, Jakob and co-workers opens the possibility that the cell survives the collapse in its NAPDH levels thanks to, rather than despite, protein oxidation.

Obviously, such ideas now need to be tested, but it will not be simple to perturb the mechanisms responsible for maintaining the balance between oxidation and reduction in cells. And even if this could be done it would be very difficult to draw meaningful conclusions from such experiments. However, the work of the Michigan group underlines the importance of studying the mechanisms that control the levels of NADPH in cells as they age. Furthermore, if the Michigan team is right, cysteine modification should prove to be a widespread and potent mechanism for the regulation of protein function, as is the case for a very similar amino acid, namely serine. Time will tell whether these attractive ideas are meaningful.
